# Flow Cytometry Evaluation of Blood-Cell-Bound Surface FVIII in Hemophilia A and Thrombosis

**DOI:** 10.3390/cells14020073

**Published:** 2025-01-08

**Authors:** Anjud Al-Mohannadi, Reem Mohammed Yahia, Hani Bibawi, Che-Ann Lachica, Watfa Ahmed, Igor Pavlovski, Giusy Gentilcore, Elkhansa Elbukhari Elgaali, Anila Ejaz, Areeg Ahmed, Mohammed Elanbari, Zainab Awada, Mohammed J. Al-Kubaisi, Muhammad Elnaggar, Ayman Saleh, Chiara Cugno, Sara Deola

**Affiliations:** 1 Research Department, Sidra Medicine, Doha P.O. Box 26999, Qatar; 2 College of Health and Life Sciences, Hamad Bin Khalifa University, Doha P.O. Box 34110, Qatar; 3 Pathology Department, Sidra Medicine, Doha P.O. Box 26999, Qatar; 4 Hematology-Oncology Clinic, Sidra Medicine, Doha P.O. Box 26999, Qatar

**Keywords:** flow cytometry, factor VIII, hemophilia A, leukocytes, monocytes, coagulation

## Abstract

Hemophilia A (HA) is associated with FVIII coagulation insufficiency or inactivity leading to excessive bleeding. Elevated FVIII, on the contrary, is associated with thrombophilia, thrombosis, myocardial infarctions, and stroke. Active FVIII (aFVIII) uses its C2 domain to bind to blood cells’ membranes, consequently carrying out its coagulative function. We developed a reliable flow cytometry (FC) method for FVIII detection that can be utilized for assessing surface-bound FVIII on leukocytes in different coagulation/clinical states; we analyzed 49 pediatric subjects, encompassing patients with HA, other coagulopathies, venous thrombosis, and normal coagulation. Interestingly, the total leukocyte surface FVIII showed a declining trend across thrombosis, normal, and hypo-coagulation states. As expected, the leukocytes of HA patients displayed significantly lower levels of cellular-surface FVIII in comparison to patients with thrombosis. However, no significant correlation was observed between circulating levels of FVIII in plasma and the levels of FVIII bound to leukocytes, indicating that the differences in FVIII surface binding are not directly proportional to the availability of FVIII in the circulation and suggesting a specific binding mechanism governing the interaction between FVIII and leukocytes. Intriguingly, when analyzing the distinct blood subpopulations, we observed that surface FVIII levels were significantly elevated in classical monocytes of thrombosis patients compared to HA patients, healthy controls, and patients with other coagulopathies. Our study highlights the reliability of our FC platform in assessing FVIII abundance on leukocytes’ membranes across coagulation states. Monocytes, particularly in cases of thrombosis, exhibit active binding of FVIII on their surface, suggesting a potential role in the pathophysiology of thrombosis that requires further investigation.

## 1. Introduction

Hemophilia A (HA) is a genetic bleeding disorder caused by the deficiency or inactivity of coagulation factor VIII (FVIII). Hypercoagulability or thrombophilia, in contrast, is clinically characterized as an increased tendency to develop thrombosis, which can be either acquired or inherited. Elevated FVIII levels were observed to be associated with thrombophilia, thrombosis, myocardial infarction, and stroke [1,2,3,4]. The structural properties and membrane-binding features of the FVIII protein are described in the literature [5,6,7].

FVIII maintains a covalent link with von Willebrand Factor at steady state and as the coagulation cascade is activated, active FVIII (aFVIII) utilizes its C2 domain to bind to the plasma membrane of platelets and other blood cells, consequently carrying out its coagulative function.

We developed a reliable flow cytometry (FC) method to detect FVIII, which can be utilized to quantify FVIII levels both on the cell surface membrane and intracellularly, within the cytoplasm of cells [8,9]. Leukocyte abundance is reported to be associated with thrombotic events; however, this phenomenon lacks a clear mechanistic explanation [10,11]. In this study, we aimed to utilize FC to evaluate the percentage of aFVIII bound on leukocytes, including lymphoid and myeloid blood subsets in various clinical conditions characterized by bleeding or thrombosis. The primary objective of this study was to evaluate whether the presence of surface-bound aFVIII could be correlated with the coagulation states.

## 2. Materials and Methods

The methodology of the study involved utilizing FC to measure the levels of cellular-surface FVIII in leftover blood samples from a variety of patients visiting the hospital.

### 2.1. Study Cohort

The study cohort comprised leftover blood samples from 49 pediatric subjects with a median age of 6 years, and a male-to-female ratio of 3:2. The evaluation involved four distinct groups, each encompassing patients with different conditions. These conditions included patients with HA (n = 13); other coagulopathies: von Willebrand Disease (vWD)/hemophilia B (HB)/unexplained menorrhagia/bleedings (n = 11); venous thrombosis (n = 13); and patients with no coagulopathies/healthy controls (n = 12) (Table 1).

### 2.2. Differential Leukocyte Count

Firstly, differential blood counts of the received leftover blood samples were used to assess cellular composition. The clinical-grade Sysmex XN-1000™ Hematology Analyzer was used to perform a clinical-grade leukocyte count in order to assess the quality of the samples received. Samples that were clotted or not evaluable for leukocytes were excluded from the FC analysis (Figure S1). All samples were received within 48 h of the blood draw.

### 2.3. Cellular-Surface FVIII FC Analysis in Total Leukocyte and Leukocyte Subpopulations

Suitable samples were treated with blood cell lysis according to the manufacturer’s procedures for VersaLyse™ Lysing Solution (Beckman Coulter, Paris, France). PBMCs were then washed twice with 3 mL of phosphate-buffered saline (PBS) before proceeding to FC staining. Viability staining was performed using Zombie UV™ fixable dye (BioLegend, San Diego, CA, USA), followed by the labeling of cells with anti-FVIII antibodies (clone GMA8024, Green Mountain Abs, San Francisco, CA, USA) utilizing Zenon labeling technology and the GMA8024 clone, as previously described [8]. The amount of antibody was titrated on 3 PBMCs leftovers (Figure S3a) with increasing FVIII Ab concentrations (0.25, 0.5, 1, 2, and 4 µg). The Zenon label amount was proportionally increased, as per the manufacturer’s instructions (5 µL of stain for each µg of Ab). FVIII positivity was calculated by subtracting IgG MFI from the FVIII MFI values. Additionally, a comprehensive immune phenotype panel was used to examine markers for lymphocytes, granulocytes, and monocytes, including CD3, CD4, CD8, and CD19 for lymphocytes, CD14 and CD33 for monocytes, and CD16 for granulocytes (BioLegend, San Diego, CA, USA). FC data were acquired through BD FACSymphony A5 Cell Analyzer and Cytek Aurora flow cytometers.

### 2.4. Gating Strategy

To gate the cellular subpopulations of leukocytes, we used a logical and strict FC gating approach starting from physical parameters of forward and side scatter to gate all leukocytes, followed by gating single cells and viable cells, as illustrated in Figure S2. For lymphoid subsets, T-lymphocytes were gated as CD3^+^CD4^+^ (helper T cells) and CD3^+^CD8^+^ (cytotoxic T cells). B-lymphocytes were gated as CD3^−^CD19^+^. For myeloid subsets, classical monocytes were gated as CD33^+^CD14^+^. Granulocytes were gated as CD16^+^. The cellular-surface-bound FVIII percentage was measured for total leukocytes and the various leukocyte subsets. FVIII positivity was calculated by subtracting the background noise from the IgG isotype control (Figure S2). Leukocyte cell subpopulations with a frequency < 1% of total live leukocytes were excluded from the analysis due to the low reliability of FVIII measurement.

### 2.5. Plasma FVIII Measurements

Plasma FVIII levels were measured with one-stage clotting assays in the Sidra Pathology Department according to clinical standard procedures. Briefly, patients’ blood was centrifuged immediately after reception at 3000 rpm for 10 min, and plasma was aliquoted and stored frozen at −80 °C before the final analysis. The time interval between the blood draw and the processing of the sample never exceeded 8 h.

### 2.6. Data Analysis

FlowJo™ v10 software was used for the FC analyses. GraphPad Prism software (version 9.2.0) and R (version 4.3.2) were used for statistical data analyses. Surface FVIII percentage on total blood cells was correlated with plasma FVIII levels using Pearson’s test. The FVIII percentage [mean ± standard error mean (SEM)] was plotted and compared across different coagulation conditions using one-way ANOVA, followed by Dunnett or Tukey’s post hoc test. A two-sided *p* < 0.05 was considered statistically significant. FVIII percentage was also associated with each coagulation condition (yes/no) using logistic regression, and the odds ratio (OR) [95% confidence interval (CI)] was reported. The receiver operating characteristic (ROC) curve was plotted, and the area under the ROC curve (AUC) metric was used to evaluate the prediction performance of the regression model.

## 3. Results

### 3.1. Description of the Study Cohort

The study cohort (n = 49 patients) and the different coagulation states are shown in Table 1 and in more detail in Supplementary Table S1. The extremes (hyper- and hypo-) of the coagulation states in this study are venous thrombosis and HA.

### 3.2. Cellular-Surface and Plasmatic FVIII Percentages in Total Leukocytes in Different Coagulation States

We analyzed each sample for lymphoid and myeloid markers and for cell-surface-bound FVIII.

**Figure 1:**
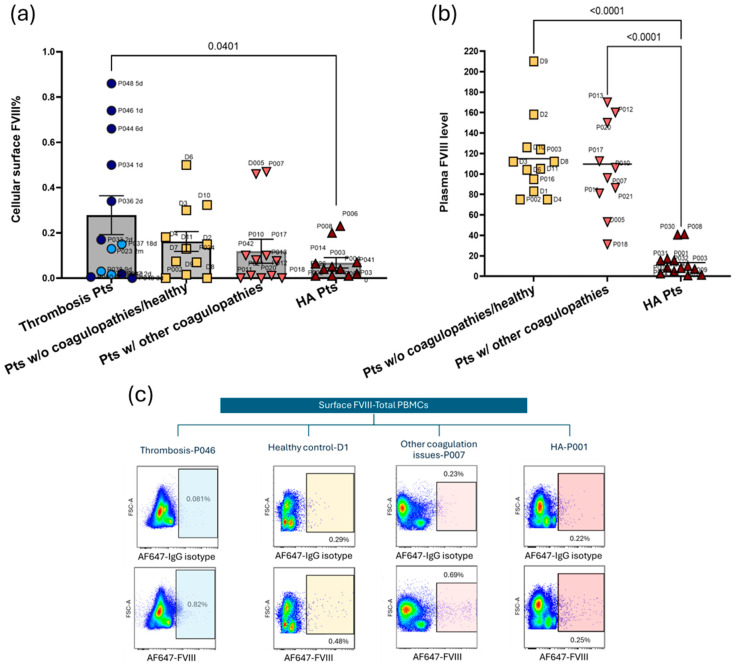
PBMC-surface FVIII by FC and in plasma. (**a**) FC analyses of surface FVIII in total PBMC were performed in the four different coagulation states (thromboses, no coagulopathies, non/HA coagulopathies, and HA). In the first column, early-onset thromboses (<1 week of onset and therapy) are represented by dark blue points and late-onset thromboses (>1 week of onset and therapy) are represented by light blue points with the day of onset (e.g., 5d = onset 5 days before measurement). (**b**) Plasma FVIII % levels were measured in parallel clinical analyses (one-stage clotting assay) for a subgroup of patients with no coagulopathies, non/HA coagulopathies, and HA. Two-sided *p*-values were derived from ANOVA followed by Dunnett or Tukey’s post hoc tests. (**c**) FC dot plots of surface FVIII on total PBMCs in 4 representative samples of the 4 coagulation states. Top line shows IgG staining, while bottom line shows FVIII staining in 4 different subjects.

The total leukocyte surface FVIII percentage showed a declining trend across thrombosis, normal, and hypo-coagulation states, and the FVIII on HA patients’ leukocytes was significantly lower in comparison with thrombosis patients’ leukocytes (0.067% versus 0.278%, *p* = 0.0401, Figure 1a). Interestingly, when correlating the results with clinical features in the thrombosis group, we noticed that patients with more recent thromboses (<1 week) trended to a higher FVIII expression (see Figure 1a column “Thrombosis Pts” and Figure S4).

The flow cytometry results were correlated with plasma FVIII measurements, performed for diagnostic purposes in a subset of the cohort (n = 36, including subjects with no coagulopathies, non/HA coagulopathies, and HA). As expected, patients with HA exhibited significantly lower plasma FVIII levels in comparison to patients with other conditions (*p* < 0.0001) (Figure 1b).

**Figure 2:**
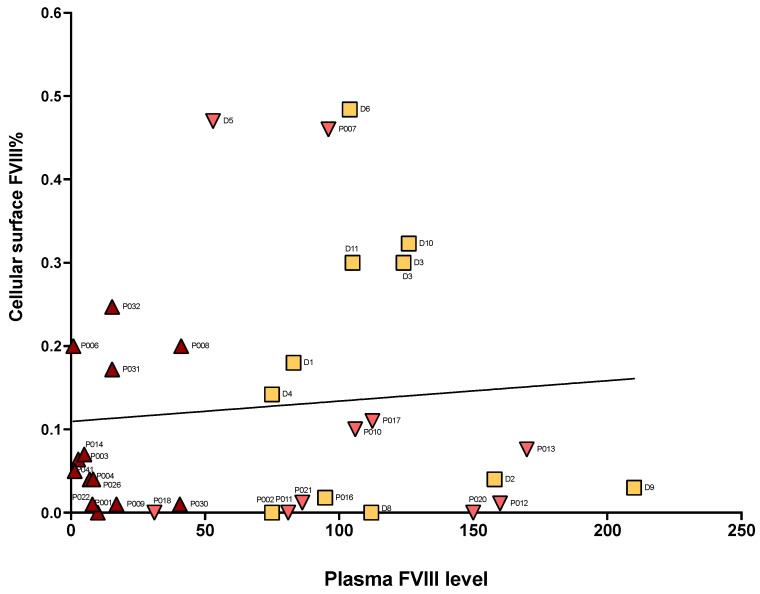
Correlational analysis. Correlational analysis of surface FVIII percentage measured by FC on total PBMCs versus plasmatic FVIII levels (one-stage clotting assay) in a subgroup of patients performing both tests in parallel (dark red = HA patients, pink = coagulation/bleeding-issues patients, and yellow = noncoagulation issues patients/normal controls). Pearson R-squared = 0.0093, *p* = 0.58.

Nevertheless, when comparing plasmatic and leukocyte-bound FVIII levels via Pearson correlational analysis, we were unable to identify any significant correlation (Figure 2). This suggests that the variations in cell-surface-bound FVIII on the membrane of leukocytes are not directly proportional to the availability of FVIII in the blood circulation, and rather suggests the existence of a specific binding mechanism.

### 3.3. Cellular-Surface FVIII in Myeloid and Lymphoid Subpopulations of Leukocytes

**Figure 3:**
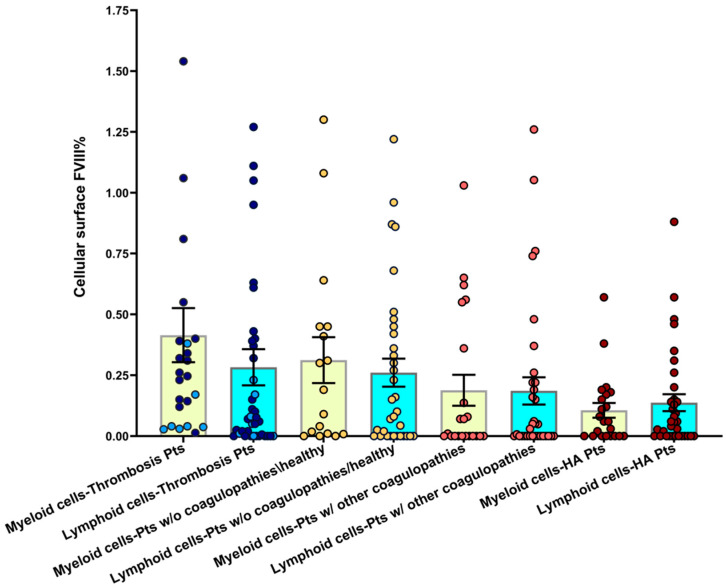
Surface FVIII percentage on myeloid versus lymphoid blood subpopulations. Bar graphs show the mean ± SEM of surface FVIII% in myeloid (CD14^+^/33^+^ monocytes and CD16^+^ granulocytes) versus lymphoid (CD3^+^ T and CD19^+^ B lymphocytes) blood leukocytes subsets across the four coagulation states. In the first 2 bars, representing thrombosis patients, early-onset thrombosis (<1 week of onset and therapy) is represented by dark blue points and late-onset thrombosis (>1 week of onset and therapy) is represented by light blue points. See Figure S2 for gating strategy in PBMC subpopulations.

Subsequently, we analyzed the surface-bound FVIII levels in different blood subpopulations. In the case of normal coagulation and thrombosis, lymphoid subpopulations (T- and B-lymphocytes) generally had slightly lower surface FVIII levels (mean = 0.27%) than myeloid cells (mean = 0.38%) (Figure 3). This result is consistent with the abundance of FVIII protein in myeloid cells that we described previously at the intracellular level [8].

**Figure 4:**
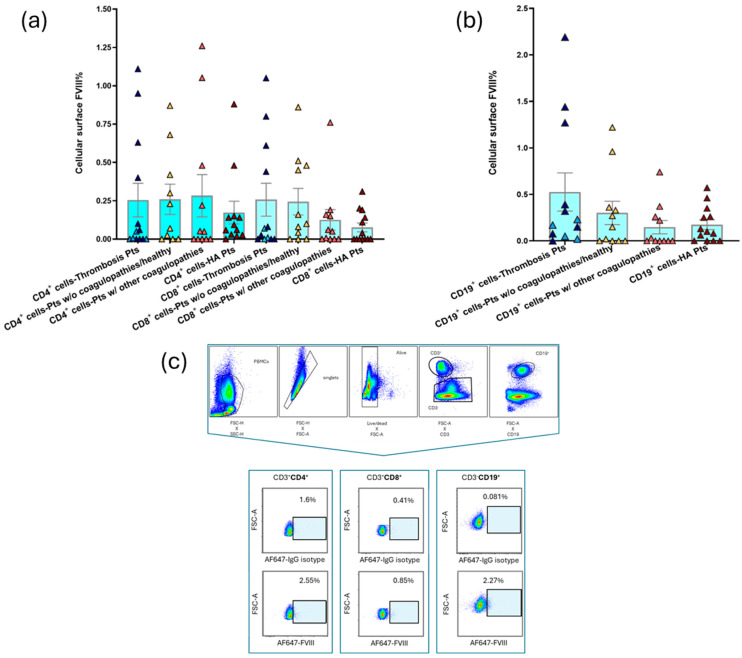
Surface FVIII percentage on lymphoid blood cells. Bar graphs show the mean ± SEM of surface FVIII% in (**a**) CD4^+^ and CD8^+^ T lymphocytes and (**b**) CD19^+^ B lymphocytes in the different coagulation states. In the columns representing thrombosis patients, early-onset thrombosis (<1 week of onset and therapy) is represented by dark blue points and late-onset thrombosis (>1 week of onset and therapy) is represented by light blue points. (**c**) FC gating strategy and surface FVIII on T and B lymphocyte subpopulations in a representative thrombosis sample (P046). See Figure S2 for gating strategy in PBMC subpopulations.

The analyses of B and T lymphoid subsets across the four coagulation states showed the lowest surface FVIII percentages in HA samples, although without significant differences across the different coagulation conditions (Figure 4).

**Figure 5:**
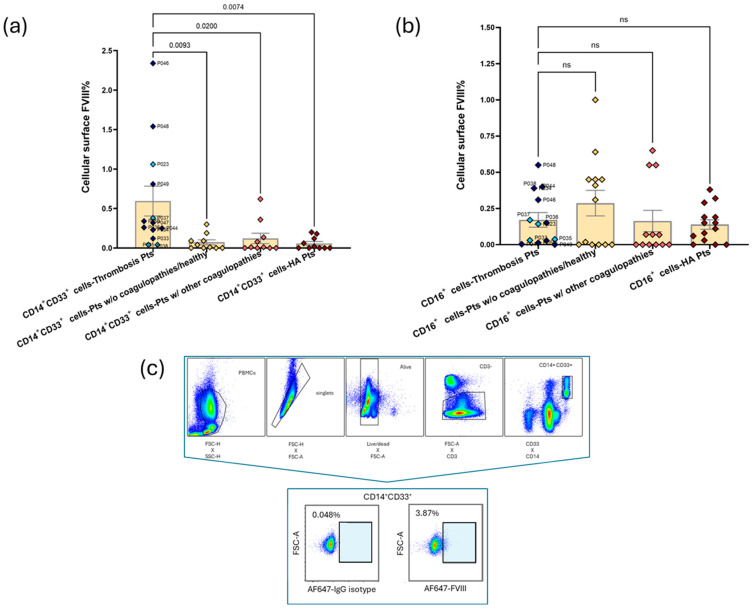
Surface FVIII percentage on myeloid blood cells. Bar graphs show the mean ± SEM of surface FVIII% in (**a**) CD14^+^/CD33^+^ classical monocytes and (**b**) CD16^+^ granulocytes. In the columns representing thrombosis patients, early-onset thrombosis (<1 week of onset and therapy) is represented by dark blue points and late-onset thrombosis (>1 week of onset and therapy) is represented by light blue points. Moreover, in the same columns, patient codes are highlighted for an easy reference to the clinical annotations in Table 1. A two-sided *p*-value was derived from ANOVA followed by Dunnett post hoc test. (**c**) FC gating strategy and dot plots of surface FVIII on classical monocytes of a representative thrombosis sample (P046). See Figure S2 for gating strategy in PBMC subpopulations.

However, when analyzing myeloid subpopulations, in CD14^+^/CD33^+^ classical monocytes, we observed a significant difference in the percentage of surface-bound FVIII levels in thrombosis patients versus HA patients (0.595% versus 0.058%, *p* = 0.0074), healthy subjects (0.07% versus 0.595%, *p* = 0.0093), and patients with other coagulopathies (0.12% versus 0.595%, *p* = 0.02) (Figure 5a). No significant differences were found in surface FVIII on granulocytes (Figure 5b).

We repeated the measurements using the absolute number of cells and FVIII expressing cells, instead of percentages, obtaining the values by multiplying patients’ WBC by the relative % of cell subsets and FVIII subset-specific % (Figure S5). This analysis showed no correlation between WBC and subset abundance and clinical status. The FVIII absolute number of expressing cells was also not correlated with clinical status, although a trend of more abundant WBC and monocytes was noted in thrombosis patients. These measurements likely underpin the limitation of a small-sized cohort and the use of leftovers, resulting in high inter-sample variability that might be less visible when dealing with percentages rather than cell numbers.

### 3.4. Cellular-Surface FVIII as a Prediction Tool for Thrombosis and Other Coagulation Disorders

We were intrigued by the association between classical monocyte surface FVIII and thrombosis, since monocytes are pathologically relevant for the thrombotic mechanism and their abundance is linked to thrombus formation.

Monocytes expose their cellular surfaces for coagulation factor complex assembly, release tissue factor, and (similar to neutrophils) produce extracellular traps (ETs) that facilitate blood cell and coagulation factor (such as FVIII and vWF) adherence at the site of thrombus formation [12].

**Figure 6:**
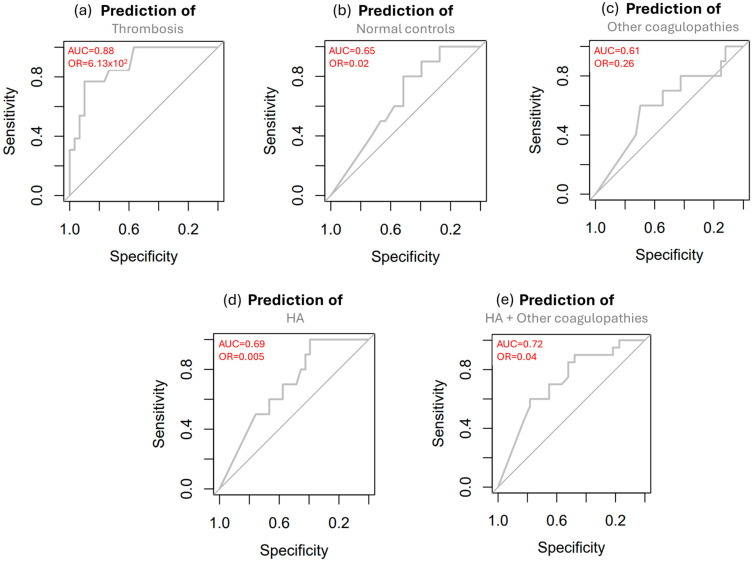
Prediction of clinical coagulation states. Graphs display prediction models of (**a**) thrombosis, (**b**) normal controls, (**c**) other coagulopathies, (**d**) HA, and (**e**) HA + other coagulopathies. Each coagulation state is predicted vs. all other states, based on the percentage of CD14^+^ CD33^+^ monocyte surface aFVIII, using binomial regression models. The resulting ROC curves are shown with the area under the curve (AUC) and the odds ratio (OR). Other parameters are thrombosis = 95% CI: (10.20–2.48 × 10^5^); *p* = 0.01, normal controls = 95% CI: (1.35 × 10^−5^–1.08); *p* = 0.20, other coagulopathies = 95% CI: (3.05 × 10^−3^–2.06); *p* = 0.38, HA = 95% CI: (6.83 × 10^−7^–0.73); *p* = 0.14, and HA plus other coagulopathies = 95% CI: (3.35 × 10^−4^–0.61); *p* = 0.09.

Thus, we sought to test whether monocyte surface FVIII levels enable the prediction of the patient’s clinical coagulation state. Binomial regression analyses showed that monocyte surface FVIII is powerful in predicting thrombosis (AUC = 0.88, *p* = 0.01) with respect to all other clinical states (Figure 6).

When we repeated the analysis using the absolute number of FVIII-expressing monocytes, instead of the percentage, we obtained similar results with a prediction of thrombosis with an AUC = 0.86, although with a *p*-value of 0.07 (Figure S6).

## 4. Discussion

The coagulative function of FVIII has been associated with its ability to adhere to cell membranes; nevertheless, there is no mechanistic data on FVIII adhering to circulating leukocytes. Using our novel FVIII FC assay, we correlated the abundance of FVIII on the surface of blood leukocytes in various coagulation situations, such as thrombosis, normal coagulation, and hypo-coagulation.

We successfully proved the reliability of our FC assay to detect even minimal quantities of FVIII on cellular surfaces. This is facilitated by the specificity of the Zenon technology used in the assay to label anti-FVIII antibodies [8]. Using this assay, we showed evidence of a logical trend of FVIII binding onto the cellular surface of different blood subpopulations in the various coagulation conditions. In general, FVIII bound more to leukocyte surfaces in thrombosis, particularly in active thrombosis (with recent onset and <1 week of therapy), followed by normal coagulation states, and was less abundant in bleeding states. Thrombotic events displayed significantly higher leukocyte surface FVIII with respect to HA. This finding aligns with the prior observations that patients with leukocytosis and a high monocyte count have an increased risk of venous thrombosis [10,11].

We could not show a significant association between plasmatic and leukocyte-bound FVIII. This lack of direct association may enforce the hypothesis of a specific mechanism for FVIII binding, rather than a passive absorption of FVIII on leukocytes, directly proportional to blood availability.

Leukocytes, mainly active neutrophils, and monocytes play a vital role in thrombosis where they accumulate during thrombus formation and produce extracellular traps (ETs, i.e., extracellular DNA fibers) and promote coagulation through increasing the adhesion between blood cells and coagulation factors like FVIII and vWF [13]. Additionally, activated monocytes release tissue factor, leading to further coagulation cascade activation and, therefore, to a reiterated involvement of FVIII in the thrombotic event [14].

Consistently, we showed that monocytes had a substantially higher abundance of surface FVIII in thrombosis patients compared to all other coagulation states.

Importantly, monocytes are a primary extrahepatic source of FVIII [15,16]. This may suggest that monocytes produce and secrete FVIII and carry part of the secreted protein on their surface, directing it into the thrombus formation site. Alternatively, they actively bind the aFVIII from the plasma pool.

The coordinate expression of tissue factor and FVIII on monocyte surface places these cells at the crossroads of the intrinsic and extrinsic coagulation pathways, implying a pivotal role in the amplification of the coagulation cascade at the site of thrombosis that might be underestimated so far.

These hypotheses were not explored in this project as they are beyond the scope of the article.

The limitations of our study include the small sample size and the use of leftovers instead of dedicated blood samples, with both factors leading to a consequent high inter-sample variability.

In conclusion, our results seed the hypothesis that active FVIII may have a monocyte-mediated role in thrombosis that is worth exploring in monocyte-mediated diseases such as atherosclerosis, tumor metastasis, and liver fibrosis [17,18,19,20,21] and in general thrombotic events.

Finally, the statistical models assessing the potential of FVIII to predict the different coagulation conditions showed that FVIII has considerable potential to distinguish between thrombosis and other coagulation states. These findings suggest that FVIII detected by FC can be a predictor of thrombosis.

## 5. Conclusions

In this pilot study, we showed that our FC platform accurately measures FVIII abundance on the membranes of blood cells across varying coagulation states. Our data suggest that the abundance of FVIII on blood cell surfaces may be predictive of thrombosis. Furthermore, CD14^+^/33^+^ monocytes actively bind aFVIII on their surface during thrombosis, suggesting a potential role in the pathogenesis of the condition through this mechanism.

## Figures and Tables

**Table 1:** Patient cohort with patient characteristics. For detailed clinical annotations, see Supplementary Table S1.

Study Cohort				
Patient Coagulation State/Category	Venous Thrombosis (VT)	Normal/No Coagulation Issues	Non-HA-Related Bleeding	HA
Number of Patients (n = 49)	13	12	11	13
Median Age, y/each category	3	12.04	11.3	2.58
Female Count/each category	2 (15.3%)	9 (75%)	8 (72.7%)	0
Male Count/each category	11 (84.6%)	3 (25%)	3 (27.27%)	13 (100%)
Common Therapy/each category	Enoxaparin	N/A	Tranexamic acid	Recombinant Factor VIII

## Data Availability

The original contributions presented in this study are included in the article/Supplementary Materials. Further inquiries can be directed to the corresponding author.

## Supplementary Materials

The following supporting information can be downloaded at: https://www.mdpi.com/article/10.3390/cells14020073/s1, Figure S1: Pre-FC Analysis Differential Leukocytes Count; Figure S2: Gating strategy scheme of FVIII’s FC analysis; Figure S3: FVIII Surface staining Validation; Figure S4: Surface bound FVIII in early versus late onset thrombosis; Figure S5: Correlation between cell numbers, FVIII and clinical classes; Figure S6: Prediction of clinical state based on the absolute number of FVIII expressing monocytes; Table S1: Detailed clinical annotations of the 49 samples.

## References

[1] Jenkins P.V., Rawley O., Smith O.P., O’Donnell J.S. (2012). Elevated Factor VIII Levels and Risk of Venous Thrombosis. Br. J. Haematol..

[2] Chang T.R., Albright K.C., Boehme A.K., Dorsey A., Sartor E.A., Kruse-Jarres R., Leissinger C., Martin-Schild S. (2013). Factor VIII in the Setting of Acute Ischemic Stroke among Patients with Suspected Hypercoagulable State. Clin. Appl. Thromb. Hemost..

[3] Vacek T.P., Yu S., Rehman S., Grubb B.P., Kosinski D., Verghese C., Eltahawy E., Shafiq Q. (2014). Recurrent Myocardial Infarctions in a Young Football Player Secondary to Thrombophilia, Associated with Elevated Factor VIII Activity. Int. Med. Case Rep. J..

[4] Brem F.L., Rasras H., El Ouafi N., Bazid Z. (2021). Acute Myocardial Infarction in a 41-Year-Old Woman due to Elevated Factor VIII: A Case Report. Pan Afr. Med. J..

[5] Dalm D., Galaz-Montoya J.G., Miller J.L., Grushin K., Villalobos A., Koyfman A.Y., Schmid M.F., Stoilova-McPhie S. (2015). Dimeric Organization of Blood Coagulation Factor VIII Bound to Lipid Nanotubes. Sci. Rep..

[6] Lü J., Pipe S.W., Miao H., Jacquemin M., Gilbert G.E. (2011). A Membrane-Interactive Surface on the Factor VIII C1 Domain Cooperates with the C2 Domain for Cofactor Function. Blood.

[7] Childers K.C., Peters S.C., Spiegel P.C. (2022). Clint Spiegel Structural Insights into Blood Coagulation Factor VIII: Procoagulant Complexes, Membrane Binding, and Antibody Inhibition. J. Thromb. Haemost..

[8] Elnaggar M., Al-Mohannadi A., Kizhakayil D., Raynaud C.M., Al-Mannai S., Gentilcore G., Pavlovski I., Sathappan A., Van Panhuys N., Borsotti C. (2020). Flow-Cytometry Platform for Intracellular Detection of FVIII in Blood Cells: A New Tool to Assess Gene Therapy Efficiency for Hemophilia A. Mol. Ther. Methods Clin. Dev..

[9] Al-Mohannadi A., Yahia R.M., Bibawi H., Lachica C.A., Ahmed W., Pavlovski I., Gentilcore G., Elgaali E.E., Ejaz A., Elanbari M. (2024). Flow-Cytometry Evaluation of Leukocytes-Bound Surface FVIII in Hemophilia A and Thrombosis. Abstract, American Society of Hematology (ASH), San Diego, US, Dec 7–10, 2024. Blood.

[10] Johnsen S.H., Fosse E., Joakimsen O., Mathiesen E.B., Stensland-Bugge E., Njølstad I., Arnesen E. (2005). Monocyte Count Is a Predictor of Novel Plaque Formation. Stroke.

[11] Barbui T., Masciulli A., Marfisi M.R., Tognoni G., Finazzi G., Rambaldi A., Vannucchi A. (2015). White Blood Cell Counts and Thrombosis in Polycythemia Vera: A Subanalysis of the CYTO-PV Study. Blood.

[12] Carobbio A., Ferrari A., Masciulli A., Ghirardi A., Barosi G., Barbui T. (2019). Leukocytosis and Thrombosis in Essential Thrombocythemia and Polycythemia Vera: A Systematic Review and Meta-Analysis. Blood Adv..

[13] Swystun L.L., Liaw P.C. (2016). The Role of Leukocytes in Thrombosis. Blood.

[14] Han Z., Liu Q., Liu H., Zhang M., You L., Lin Y., Wang K., Gou Q., Wang Z., Zhou S. (2023). The Role of Monocytes in Thrombotic Diseases: A Review. Front. Cardiovasc. Med..

[15] Zanolini D., Merlin S., Feola M., Ranaldo G., Amoruso A., Gaidano G., Zaffaroni M., Ferrero A., Brunelleschi S., Valente G. (2015). Extrahepatic Sources of Factor VIII Potentially Contribute to the Coagulation Cascade Correcting the Bleeding Phenotype of Mice with Hemophilia A. Haematologica.

[16] Elnaggar M., Al-Mohannadi A., Hasan W., Abdelrahman D., Al-Kubaisi M.J., Pavlovski I., Gentilcore G., Sathappan A., Kizhakayil D., Ali A.I. (2023). CD14^+^/CD31^+^ Monocytes Expanded by UM171 Correct Hemophilia a in Zebrafish upon Lentiviral Gene Transfer of Factor VIII. Blood Adv..

[17] Tacke F., Alvarez D., Kaplan T.J., Jakubzick C., Spanbroek R., Llodra J., Garin A., Liu J., Mack M., van Rooijen N. (2007). Monocyte Subsets Differentially Employ CCR2, CCR5, and CX3CR1 to Accumulate within Atherosclerotic Plaques. J. Clin. Investig..

[18] Gil-Bernabé A.M., Ferjancic S., Tlalka M., Zhao L., Allen D., Im J.H., Watson K., Hill S.A., Amirkhosravi A., Francis J.L. (2012). Recruitment of Monocytes/Macrophages by Tissue Factor-Mediated Coagulation Is Essential for Metastatic Cell Survival and Premetastatic Niche Establishment in Mice. Blood.

[19] Qian B.-Z., Li J., Zhang H., Kitamura T., Zhang J., Campion L.R., Kaiser E.A., Snyder L.A., Pollard J.W. (2011). CCL2 Recruits Inflammatory Monocytes to Facilitate Breast-Tumour Metastasis. Nature.

[20] Heymann F., Trautwein C., Tacke F. (2009). Monocytes and Macrophages as Cellular Targets in Liver Fibrosis. Inflamm. Allergy-Drug Targets.

[21] Karlmark K.R., Weiskirchen R., Zimmermann H.W., Gassler N., Ginhoux F., Weber C., Merad M., Luedde T., Trautwein C., Tacke F. (2009). Hepatic Recruitment of the Inflammatory Gr1^+^ Monocyte Subset upon Liver Injury Promotes Hepatic Fibrosis. Hepatology.

