# Supplementary material for: Flow Cytometry Evaluation of Blood-Cell-Bound Surface FVIII in Hemophilia A and Thrombosis
Source: Cells. 2025 Jan 8;14(2):73. doi: 10.3390/cells14020073 (PMC11764290; doi:10.3390/cells14020073)
Supplement: Supplementary file 1 [file cells-14-00073-s001.zip › cells-3354553-supplementary-final.pdf]

Supplemental Table S1. Detailed clinical annotations of the 49 samples

Sample code	Patient coagulation state/category	Age	Sex	Reason for coming to clinic	Thrombosis onset	type of thrombosis	Therapy
P023	VT	3 yrs	M	Hypoplastic left heart syndrome	2 months	CVC related, iliac/common femoral vein	Heparin
P033	VT	6 yrs	M	Tufting enteropathy (no villi)	2 days	CVC related, jugular vein	Enoxaparin
P034	VT	8yrs	M	Acute lymphoblastic leukemia	1 day	PICC line related, brachial vein	Enoxaparin
P035	VT	4yrs	F	Very Early Onset IBD in TPN	6 days	CVC related-infectious thrombus, inferior vena cava + right atrium	Enoxaparin, thrombectomy, antibiotics
P036	VT	9 months	F	Patient with multi-organ dysfunction, chronic respiratory failure, tracheostomy	2 days	CVC related, jugular vein	Enoxaparin
P037	VT	9 months	M	Post surgery complications for heart disease correction, ICU patient	18 days	CVC related, multiple sites	Heparin/enoxaparin
P038	VT	2yrs	M	Thrombosis in Budd-Chiari syndrome	9 days	Hepatic inferior vena cava, unknown causes	Heparin/enoxaparin, thrombectomy, tissue plasminogen
P044	VT	4yrs	M	Otomastoiditis	6 days	Cerebral sinus thrombosis following otitis media	Enoxaparin
P046	VT	5 yrs	M	Sepsis	1 day	Heart	Enoxaparin
P047	VT	6 mo	M	Chronic lung disease	2 days	CVC related, lower limb	Enoxaparin
P048	VT	19 m	M	Thrombosis	5 dyas	PICC line related, brachial vein	Enoxaparin
P049	VT	12 days	M	intestinal perforation	3 days	Cerebral sinus thrombosis	Enoxaparin
P050	VT	10 yrs	M	Thrombosis	1 day	Pulmonary embolism	Enoxaparin
D1	Normal/no coag.	3 mo	M	Meningitis/Check-up to rule out immunodeficiency	N/A		Antibiotics/antiviral
D2	Normal/no coag.	2 yrs 7mo	F	Infantile Osteopetrosis post bone marrow transplant	N/A		Eltrrombobag
D3	Normal/no coag.	35 yrs	F	Miscarriage	N/A		Hematemics

D4	Normal/no coag.	12 yr 2 mo	M	SPASTIC Cerebral Palsy, Rheumatic heart disease, obesity	N/A	Penicillin V, riboflavin
D6	Normal/no coag.	11 yrs 11 mo	F	Developmental and learning delay, bilateral sensorineural deafness, genetic abnormality, epilepsy, hypothyroidism	N/A	Carbama./ Levothyroxine
D7	Normal/no coag.	9 yr 1 mo	F	Epistaxis/Atopic dermatitis	N/A	N/A
D8	Normal/no coag.	31 yr 1 mo	F	Healthy/Checkup	N/A	Omeprazole
D9	Normal/no coag.	14 yr 6 mo	M	Dandy Walker syndrome	N/A	N/A
D10	Normal/no coag.	24 yr 8 mo	F	Gestational Hypothyroidism	N/A	N/A
D11	Normal/no coag.	5 mo	F	Healthy/Checkup	N/A	N/A
P002	Normal/no coag.	10 yrs 4m	F	Bilateral choanal atresia, asthma, right ectopic kidney	N/A	Albuterol, Cetirizine
P024	Normal/no coag.	30 yrs	F	Secondary Oligomenorrhea	N/A	
D5	Coag./bleeding	11 yrs 4mo	F	Menorrhagia	N/A	Tranexamic acid, norethindrone
P007	Coag./bleeding	13 yrs	F	Menorrhagia	N/A	Tranexamic acid
P010	Coag./bleeding	13 yrs	F	Type-1 Diabetes Miletus/Menorrhagia	N/A	Tranexamic acid
P011	Coag./bleeding	9 yrs	F	Nipple bleeding	N/A	Tranexamic acid
P012	Coag./bleeding	17 yrs	F	Irregular Menstruation	N/A	Tranexamic acid
P013	Coag./bleeding	17 yrs	F	Menorrhagia	N/A	Tranexamic acid
P017	Coag./bleeding	9 yrs	F	Menorrhagia	N/A	Tranexamic acid
P018	Coag./bleeding	6 mo	M	Von Willebrand disease	N/A	N/A
P020	Coag./bleeding	5 yrs	M	Von Willebrand disease	N/A	N/A
P021	Coag./bleeding	15 yrs	F	Menorrhagia	N/A	N/A
P042	Coag./bleeding	8 yrs	M	Severe hemophilia B	N/A	N/A
P001	HA	2 yr 7mo	M	Severe Hemophilia A	N/A	Recombinant factor 8
P003	HA	6 wks	M	Severe hemophilia A	N/A	Recombinant factor 8
P004	HA	2 yrs	M	Severe hemophilia A with inhibitors	N/A	Recombinant factor 8
P006	HA	23 mo	M	Severe hemophilia A	N/A	Recombinant factor 8
P008	HA	22 mo	M	Moderate Hemophilia A	N/A	Recombinant factor 8
P009	HA	8 yrs	M	Severe Hemophilia A	N/A	Recombinant factor 8
P014	HA	4 yrs	M	Severe Hemophilia A without inhibitors	N/A	Recombinant factor 8
P022	HA	17 mo	M	Severe Hemophilia A without inhibitors	N/A	Recombinant factor 8
P026	HA	4 yrs	M	Moderate Hemophilia A	N/A	Recombinant factor 8
P030	HA	14 yrs	M	Moderate Hemophilia A	N/A	Recombinant factor 8

P031	HA	15 yrs	M	Moderate Hemophilia A	N/A	Recombinant factor 8
P032	HA	15 yrs	M	Moderate Hemophilia A	N/A	Recombinant factor 8
P041	HA	2 yrs	M	Severe Hemophilia A	N/A	Recombinant factor 8

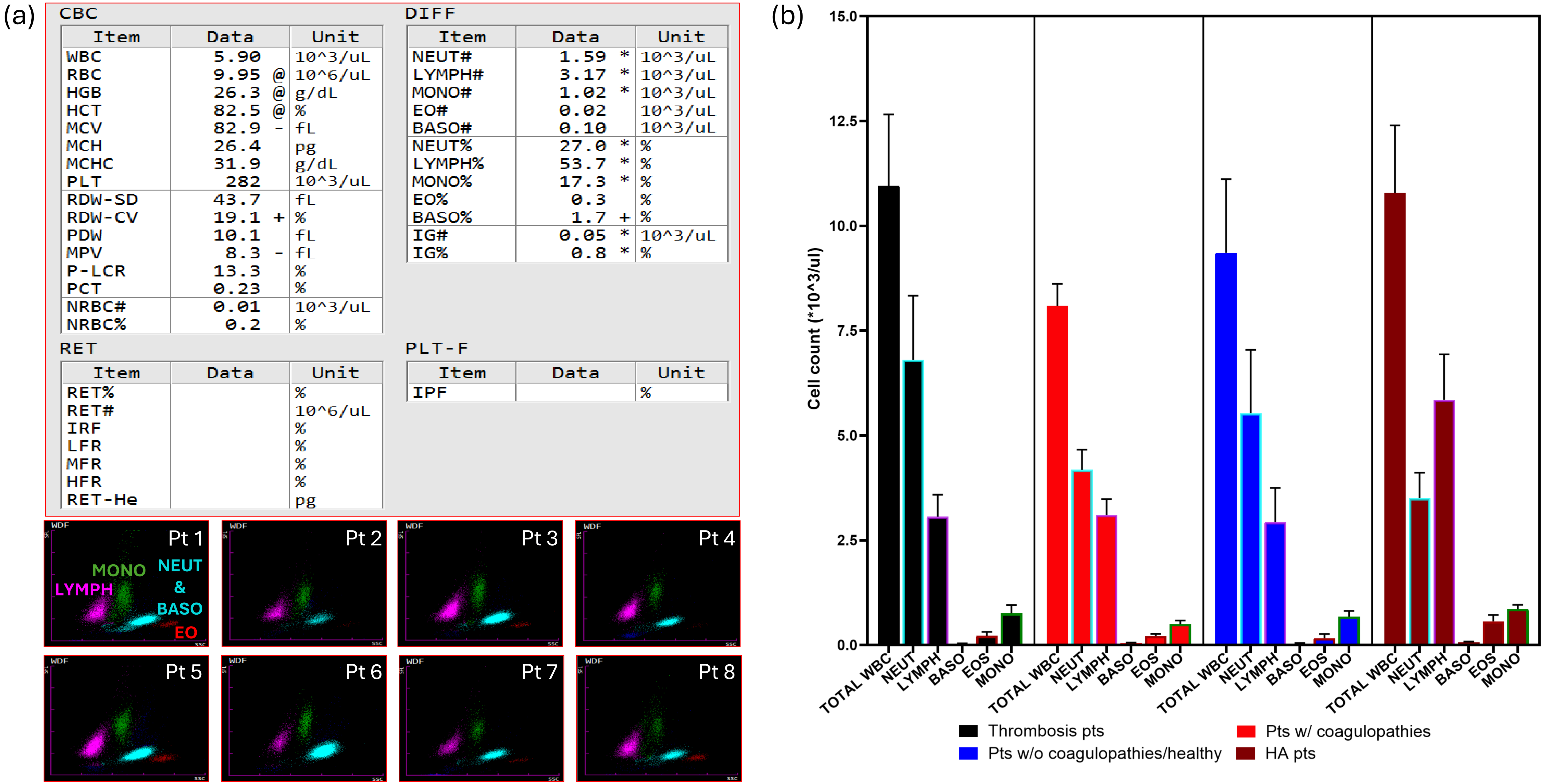

Figure S1: Pre-FC Analysis Differential Leukocytes Count. (a) Screenshot of the Sysmex XN-1000™ analysis and representative dot plots of the samples' WBC differential count. **(b)** Average leukocytes' counts of 48 blood samples in the four different coagulation states. WBC: white blood cells, NEUT: neutrophils, LYMPH: lymphocytes, BASO: basophils, EO: eosinophils, and MONO: monocytes.

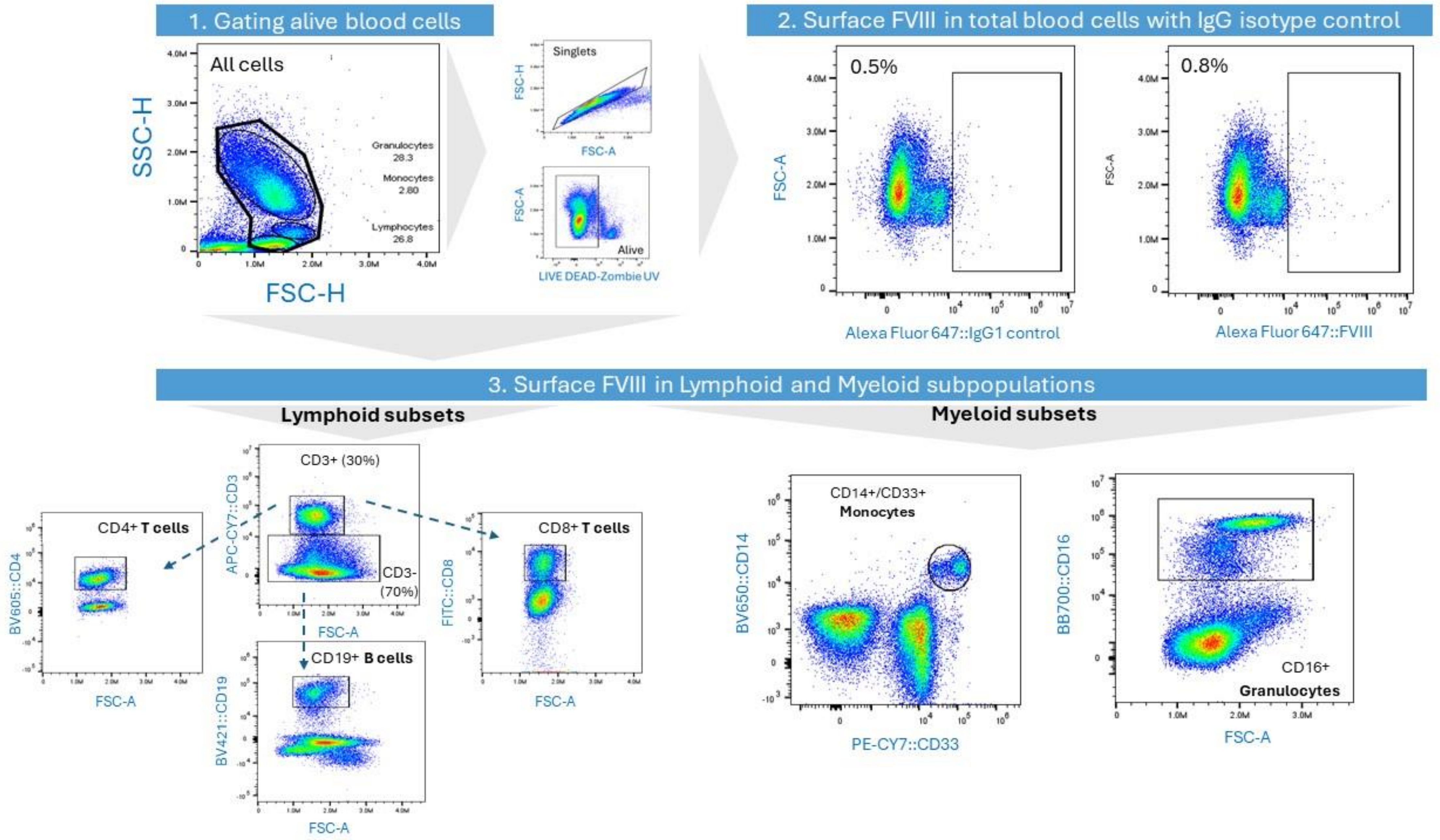

Figure S2: Gating strategy scheme of FVIII's FC analysis. 1. All cells are gated; viable single cells are selected and **2.** surface FVIII on total leukocytes is evaluated, IgG1 isotype control is used for FVIII calculations. **3.** Surface FVIII is then analyzed for the different blood lymphoid and myeloid subpopulations, selected based on the gating strategy shown (left for lymphocytes, right for myeloid cells).

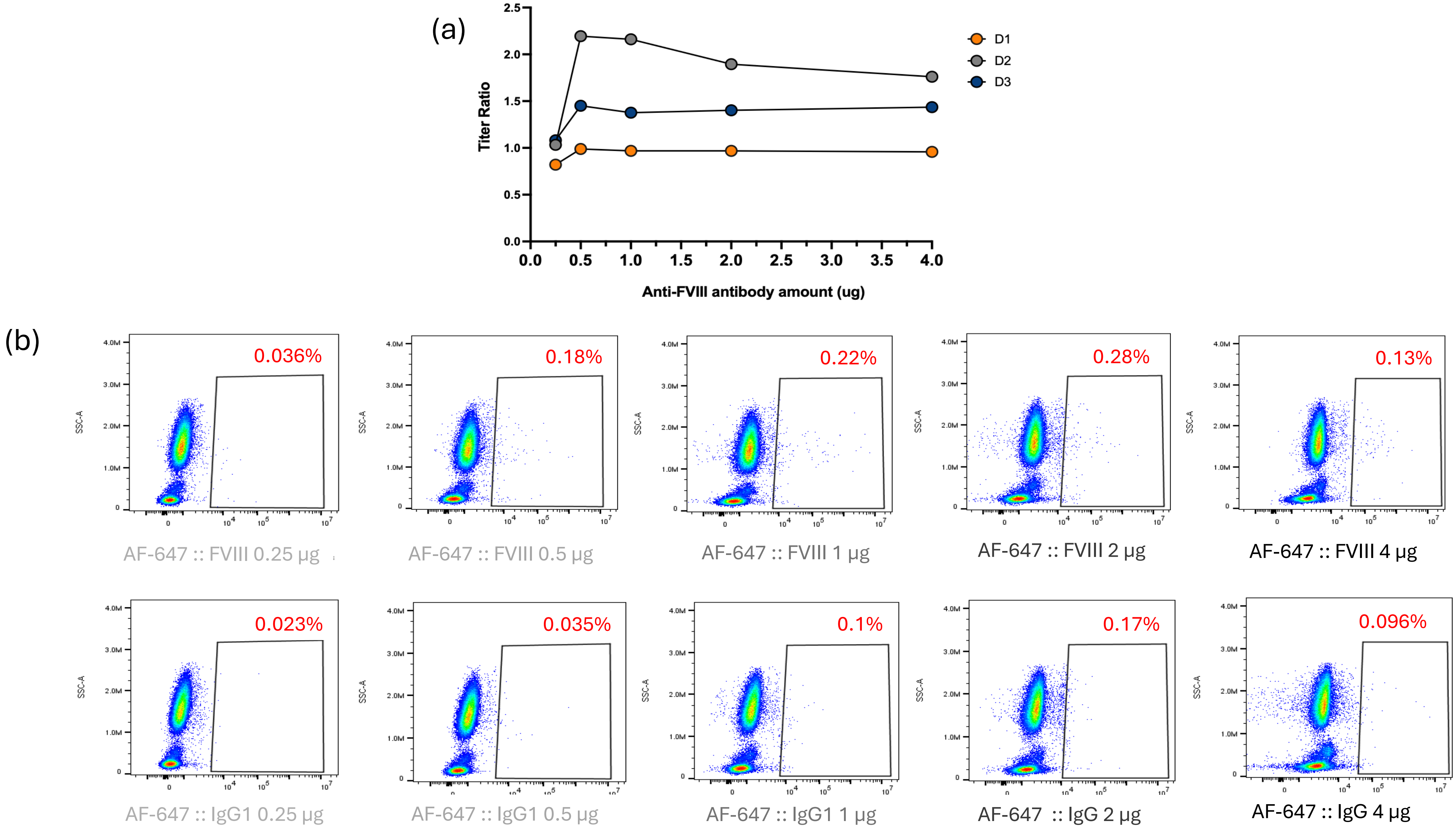

Figure S3: FVIII Surface staining Validation. Surface FVIII staining of PBMCs with different concentrations of anti-FVIII Ab (0.25, 0.5, 1, 2 and 4 µg) was performed to validate the optimal amount of antibody to be used in the FC analysis. **(a)** Antibody titration graph illustrating FVIII titer ratio with respect to the different anti-FVIII antibody concentrations. Titer ratio is calculated dividing FVIII median fluorescence by IgG median fluorescence. **(b)** Representative FC dot plots of FVIII (above) and their IgG isotype controls (below) in donor 2.

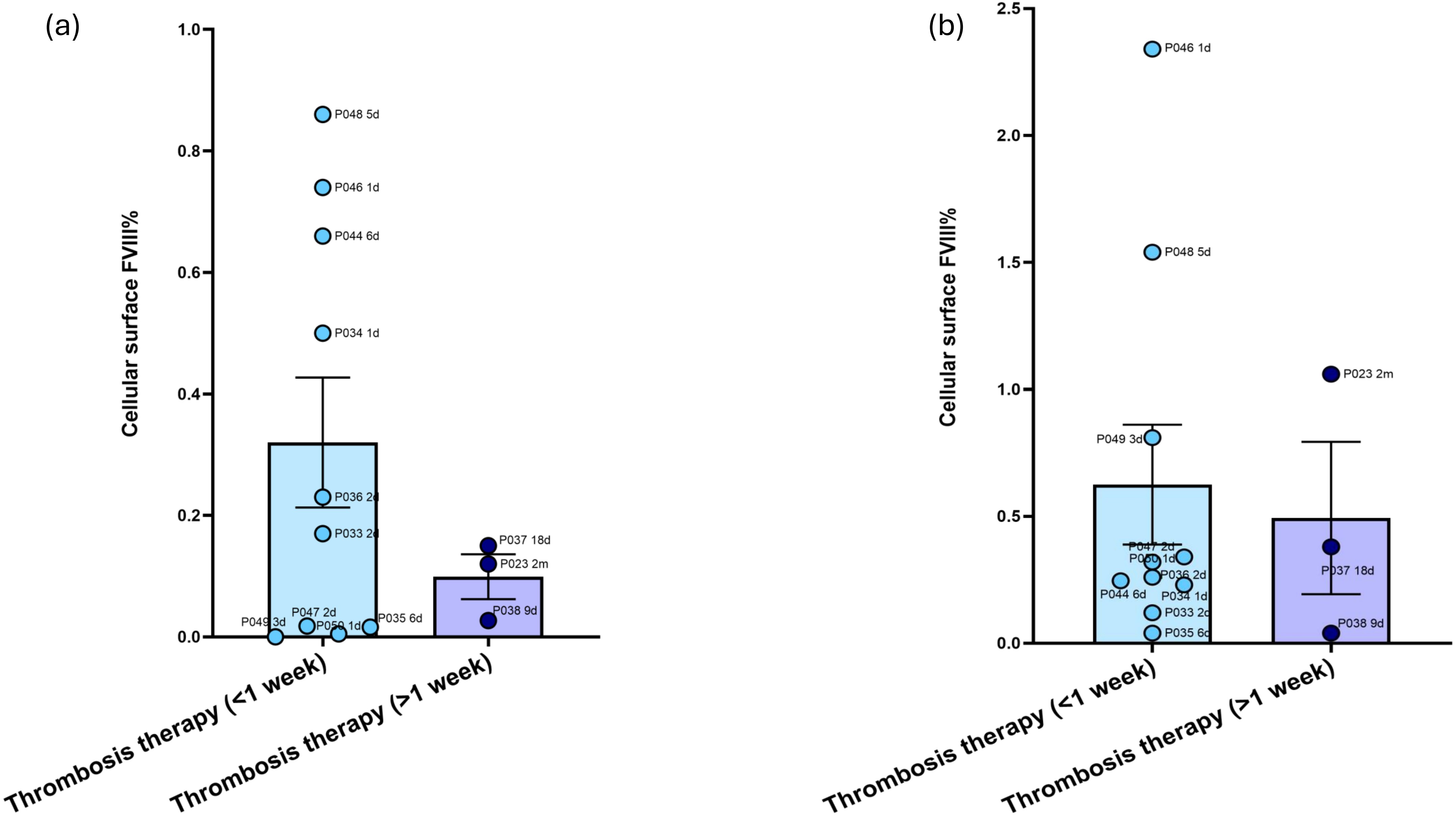

Figure S4: Surface bound FVIII in early versus late onset thrombosis. Surface FVIII in total blood cells (a) and on Monocytes (b) in samples with more recent thromboses or less treatment period (<1 week) versus (> 1 week) of thrombosis therapy.

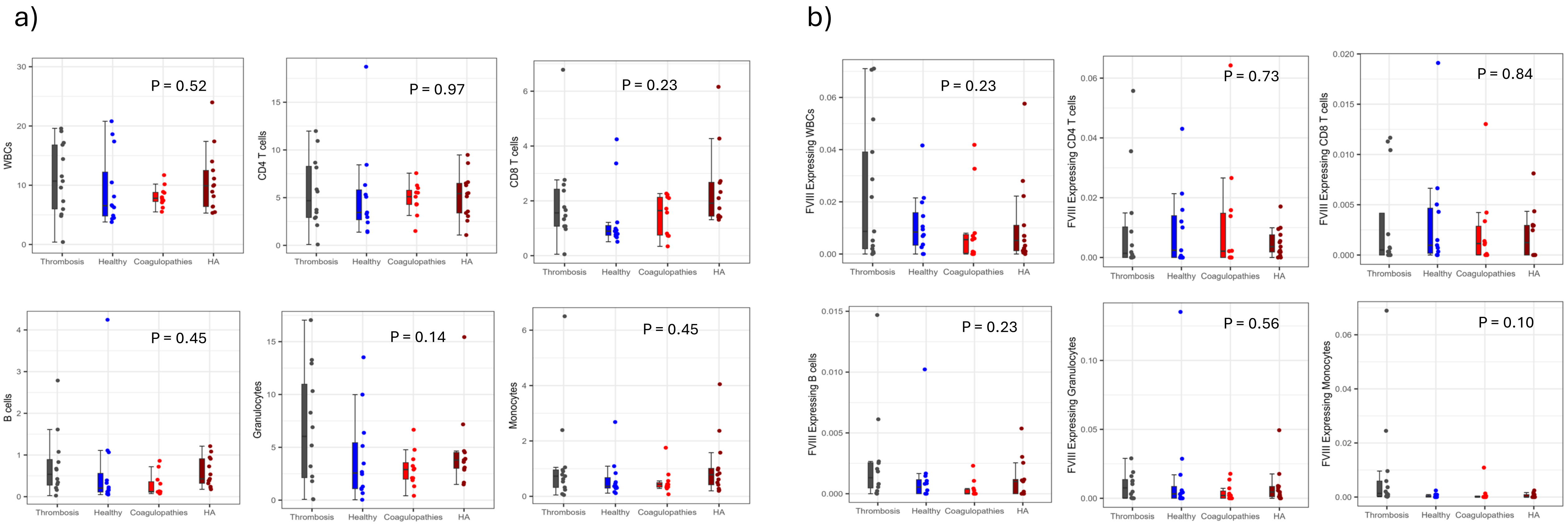

Figure S5. Correlation between cell numbers, FVIII and clinical classes. Absolute number **(a)** and absolute number of FVIII expressing **(b)** WBC and subsets were compared among the four clinical statuses using one-way ANOVA when the group variances were equal, or Welch's test when the variances differed. Absolute numbers of cells were calculated based on the flow-cytometry analyzed subpopulations, by multiplying each blood subset (see Figure S2 for logical gating derived subsets) by the absolute number of WBC for each subject (n=WBC x percentage of positive population/100).

Absolute number of FVIII positive cells in each blood subset was calculated the same way (n=absolute number of cell subset x percentage of FVIII for the same subset/100)

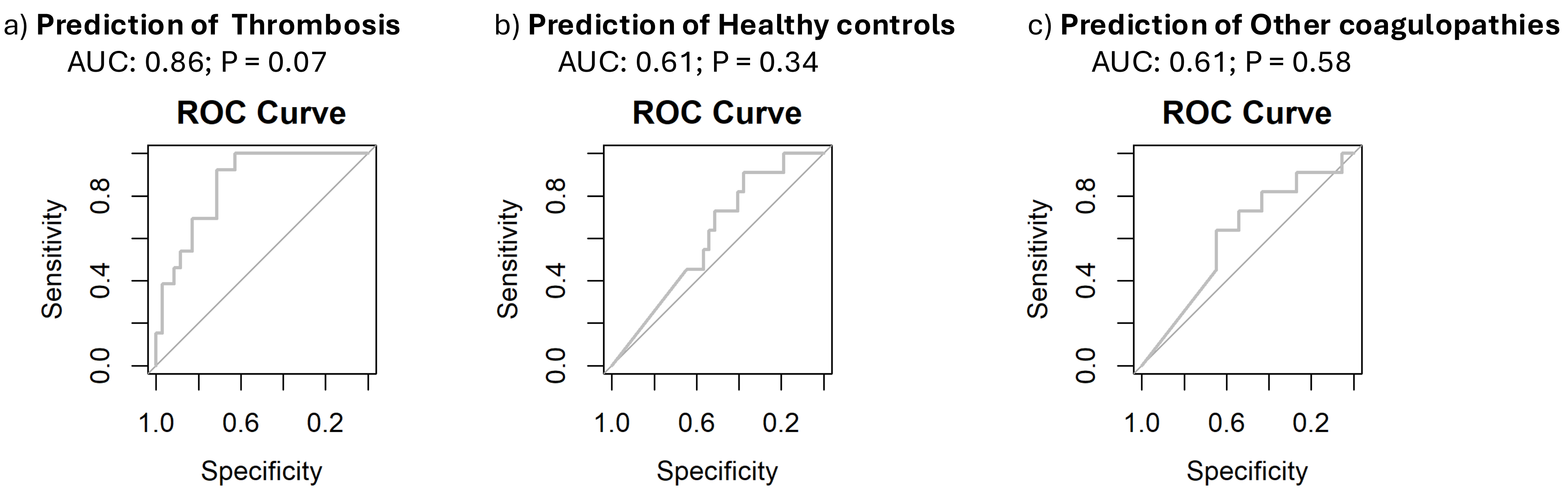

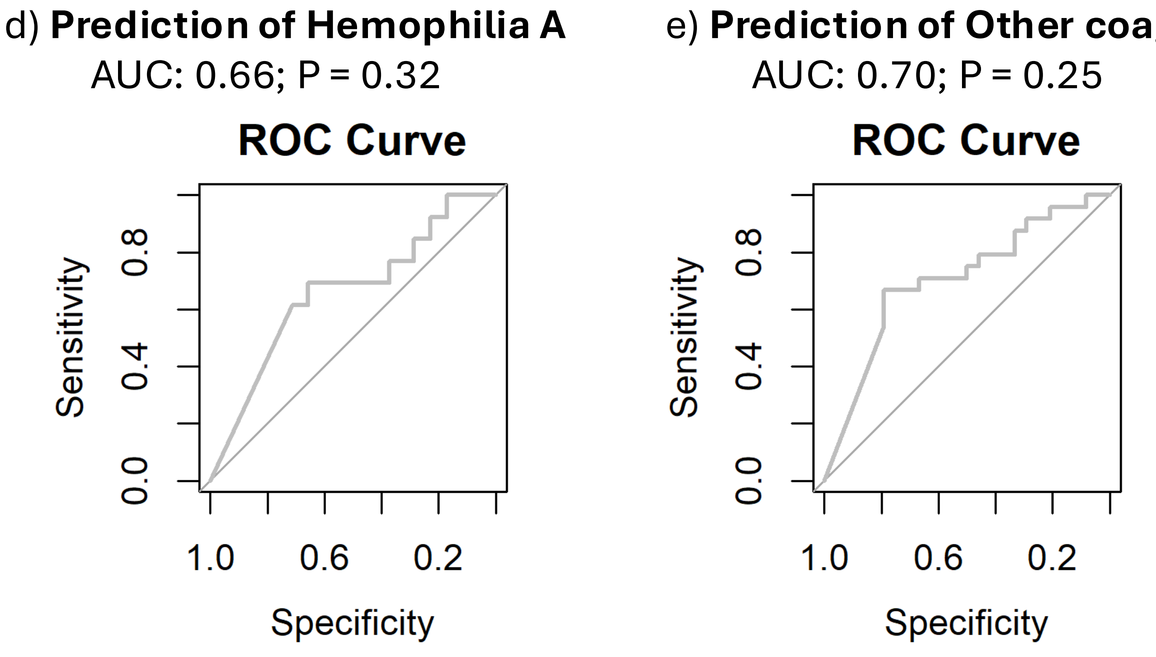

Figure S6. Prediction of clinical state based on the absolute number of FVIII expressing monocytes.
Binary logistic regression tests were performed, as for Figure 6, but based on the absolute number of FVIII expressing monocytes, rather than the %of FVIII positive monocytes. ROC curves are plotted with AUC and p values.
